# An Improved Method for Removal of Azo Dye Orange II from Textile Effluent Using Albumin as Sorbent

**DOI:** 10.3390/molecules171214219

**Published:** 2012-11-30

**Authors:** Tadashi Ohashi, Alícia M. T. Jara, Anabelle C. L. Batista, Luciana O. Franco, Marcos A. Barbosa Lima, Mohand Benachour, Carlos A. Alves da Silva, Galba M. Campos-Takaki

**Affiliations:** 1 Technical School SENAI-Paulista, Paulista 53417-710, PE, Brazil; E-Mail: tadashiohashi49@yahoo.com.br; 2 Nucleus of Research in Environmental Sciences-NPCIAMB, Catholic University of Pernambuco, UNICAP, 50050-590 Recife, PE, Brazil; E-Mails: aliciamdpa@yahoo.com.br (A.M.T.J.); bellecamarotti@yahoo.com.br (A.C.L.B.); lucianafranco@terra.com.br (L.O.F.); mablima33@yahoo.com.br (M.A.B.L.); calves@unicap.br (C.A.A.S.); 3 Network of Biotechnology, Catholic University of Pernambuco, Recife 50050-900, PE, Brazil; 4 Microbiology Department, Federal Rural University of Pernambuco, Recife 52171-900, PE, Brazil; 5 Chemical Engineering Department, Federal University of Pernambuco, Recife 50670-901, PE, Brazil; E-Mail: mbena@ufpe.br

**Keywords:** acid orange II, albumin, decolourization, toxicity, adsorption, isotherm

## Abstract

Azo dyes are generally resistant to biodegradation due to their complex structures. Acid orange II is one of the most widely used dyes in the textile industry. The influence of bovine serum albumin (BSA) in different concentrations, pH, and time of contact on Orange II was investigated using kinetics and adsorption-isotherm experiments. The results showed that the maximum colour removed from dye/albumin was 99.50% and that a stable dye-protein complex had been formed at pH 3.5 and in a proportion of 1:3 (v/v), respectively. The synthetic effluent did not show toxicity to the microcrustacean *Artemia salina*, and showed a CL_50_ equal to 97 µg/mL to azo dye orange II. Additionally, the methodology was effective in removing the maximum of orange II using BSA by adsorption at pH 3.5 which mainly attracted ions to the azo dye during the adsorption process. This suggests that this form of treatment is economical and easy to use which potentially could lead to bovine serum albumin being used as a sorbent for azo dyes.

## 1. Introduction

The release of colored wastewaters from the textile industry is a current problem encountered in developed and under developed countries all over the World, and this phenomenon is particularly notable for toxic products such as azo dye pigments which are known for their efficiency in the dyeing process. Azo dyes, which mostly consist of aromatic moieties linked together by -N=N- bonding (a type of chromophore), represent the largest class among these pollutants which are water soluble. Their release into the natural environment, mainly in an aqueous medium, is undesirable because of the adverse effect of these toxic and carcinogenic compounds on aquatic life that thus causes the destruction of ecosystems [1,2,3,4].

Currently, the textile industry uses more than 10,000 ± types of dyes, and 6,000 of these pigments are mono-azo or polyazoic azo dyes. In Brazil the textile industry accounts for 2.6% of the global demand for these azo dyes [5,6]. However, the literature reports that a significant amount of these dyes (10 to 35%) is not fixed to the fibres and the pigment can be lost, and released into the environment, which can lead to the inhibition of photosynthesis [7,8,9,10].

Various physical-chemical processes and biological methods (filtration, coagulation, precipitation, adsorption, ion exchange, and oxidation) were used in the past to remove dye coloration from wastewater. However, a lack of efficiency was observed in this process because the pollution is simply transferred from one phase to another which leads to the treatment of textile effluents being costly. The presence of heavy metals associated with the dyes may also cause chronic toxicity and human health problems [11,12,13,14,15].

Previous studies describing the use of proteins for adsorption processes focused on the physico-chemical process. Additionally, the process of membrane filtration was investigated in biomedical research using a physico-chemical process to adsorb proteins, and for their subsequent separation [16]. One must first understand the serum binding ability of the small molecules of interest, as described in detail in the literature with regard to the extraction of dyes. The diffusion of dye molecules is directly related to their size and colloidal properties [17].

Studies on filtration membranes are often applied to the movement of extracorporeal and macromolecules, such as polysaccharides or proteins, and this phenomenon has been observed in haemodialysis studies, when considering the interaction between heparin and plasmatic proteins [18,19]. Bovine serum albumin-BSA is the most abundant protein in blood plasma, and works as a transport protein for several substances, and has properties such as pH-dependent binding and interaction considering the efficiency and economic viability [20,21,22,23]. The method of vacuum filtration for the retention of synthetic dyes is described by the literature as being poor, its success being related to the extent of membrane porosity and the molecular size of synthetic dyes as prerequisites for adsorption and vacuum filtration [24,25,26].

This paper investigates the effect of pH on the adsorption process of the azo dye Orange II using bovine serum albumin (BSA), focusing on the possible interactions associated with the decolorization process, given the principle of the interaction between the dye molecules (cationic system) and albumin. The physical process of vacuum filtration was used to evaluate the decolourization of the effluent, and the complex albumin-Orange II formed, followed by evaluation of the toxicity of the decolorized effluent using the microcrustacean *Artemia salina*.

## 2. Results and Discussion

### 2.1. Influence of the Time Interaction, pH and Adsorption

In order to find the optimal contact time and albumin (BSA) concentrations, the experiments were conducted at a pH of 3.0. The relative proportions of dye to albumin were maintained (at 1:3 v/v). The results indicated that the highest contact time (90 min) of dye and albumin led to the removal of Orange II (Table 1).

**Table 1:** Removal of Orange II related to volume (azo dye/albumin) pH and time of incubation.

Assay	Azo dye/albumin concentration (g/L)	Time of incubation (min.)	pH	Orange II removal (%)
01	0.10	20	3.0	10.72
02	0.10	30	3.0	21.49
03	0.10	60.	3.0	54.06
04	0.10	90.	3.0	87.28
05	0.10	60	3.2	78.95
06	0.10	60	3.5	84.20
07	0.10	60	3.8	63.40
08	0.10	60	4.0	55.71

Parameters: Azo dye/albumin ratio (1:3 v/v); vortex at 200 r.p.m.

**Figure 1:**
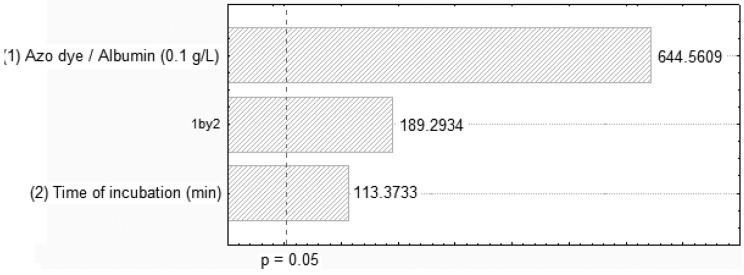
Pareto chart of standardized effects of removing Orange II related to azo dye/albumin (0.1 g/L) concentrations and time of incubation.

**Figure 2:**
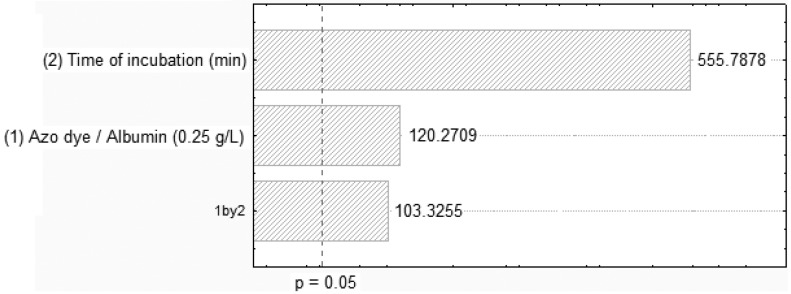
Pareto chart of standardized effects of removing Orange II related to azo dye/albumin (0.25 g/L) concentrations and time of incubation.

The effects of dye and BSA on decolorization, as well as the interaction between them, in the first 2^2^ full factorial designs are shown in Figure 1 and Figure 2. The Pareto chart clearly shows a positive correlation of the dye/albumin and the contact time on the decolorization process. Higher interaction was observed on the dye Orange II and BSA concentrations of 0.1 g/L and 0.25 g/L at 90 min of contact and pH of 3.0, respectively. The low error (0.093667) obtained from the standardised effects of adsorption by BSA validated the experimental procedure using azo dye/albumin (0.25 g/L). On the other hand, the Orange II-BSA interaction contributes in a statistically significant way to the increase in the decolorization of the effluent.

In addition, the results of our experiments show that BSA adsorption is superior to non-conventional methods, such as tangential flow filtration with membranes and bioelectrochemical systems, for the removal of azo dye with a view to totally removing the dye [27,28].

**Figure 3:**
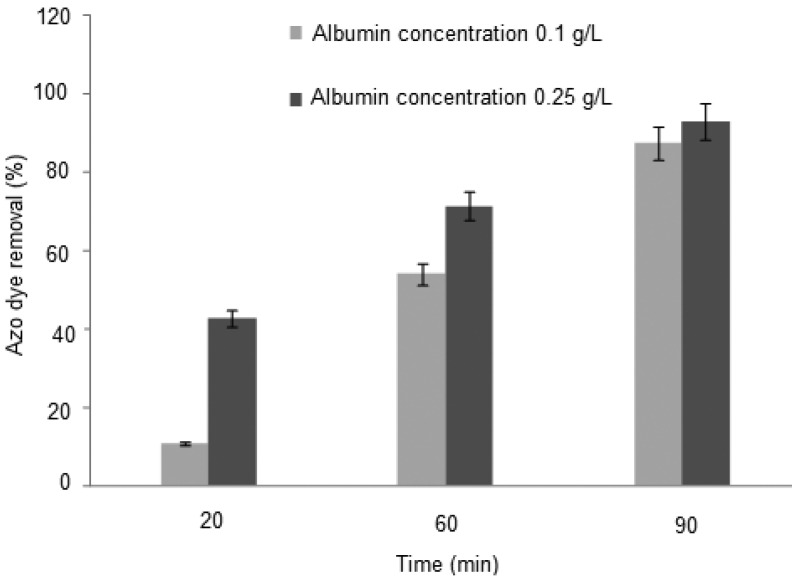
Efficiency of removing azo dye Orange II using albumin in different concentrations (0.10 and 0.25 g/L, v/v) in pH 3.0 and incubation time.

**Figure 4:**
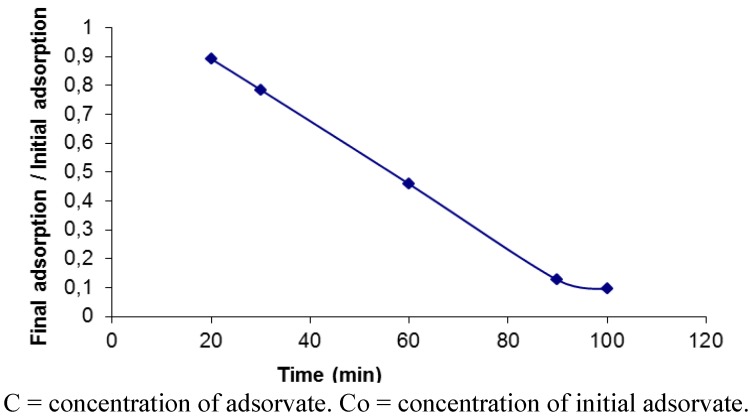
**A**dsorption of the azo dye Orange II using albumin.

The initial parameters obtained from the phenomenon of chemical precipitation of Orange II-BSA observed at both the beginning and the end by separation of and the end of separating the membrane filter are shown. For this, the interaction time was set to 20 to 90 min, the dye/albumin ratio was 1:3, and the pH was varied from 3.0 to 4.0. The pH value with the highest decolorization (92.91%) was pH 3.0 (Figure 3). The results suggest the influence of the interaction time is a very important property on the adsorption capabilities of albumin. These results are corroborated by reports in the literature that indicate that increasing the molecular volume of albumin enables more Orange II to be adsorbed [20,22,26,29]. The kinetic for adsorption of Orange II on BSA at pH 3.0 (cationic form) can be explained taking into account that the ionic attraction contributes to increasing the BSA interaction phenomena, and leads to maximizing the dye decolorization process (Figure 4).

### 2.2. Removal of Orange II in the Azo Dye/Albumin (v/v) System, and Time of Interaction

The data obtained from the experiments were analysed to evaluate the efficiency of the method (ability of BSA to remove Orange II). The effects of pH, contact time and albumin/Orange II volume ratio on Orange II removal are presented (Table 2).

**Table 2:** Interaction of the dye Orange II and albumin proportions related to concentrations maintained in the pH 3 and, contact time of 90 min.

Assay	Azo dye/albumin proportions (mL)	Orange II removal (%)
01	1:1 (25 + 25)	27.48
02	1:2 (25 + 50)	56.21
03	1:3 (25 + 75)	84.20
04	1:4 (25 + 100)	33.33
05	1:5 (25 + 125)	27.85

**Figure 5:**
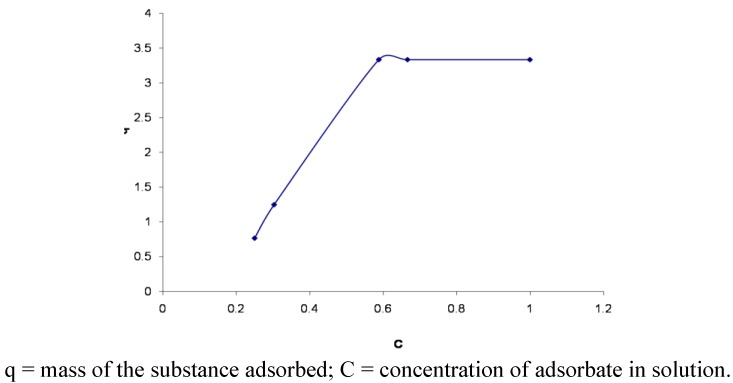
Isotherm of adsorption of azo dye orange II and albumin.

Parameters such as pH, agitation and the v/v relationship of the dye/albumin may be affected by the interaction (Table 3 and Figure 5). The efficiency of separation is independent of the average porosity of the membrane, thus providing additional support for the use of this method of adsorption. The condition that provided the greatest removal was an interaction time of 90 min, an albumin concentration of 0.25% g/L (ratio 1:3) and a pH of 3.0. Under these conditions 92.50% removal of Orange II was achieved. The methodology demonstrated here provides more effective removal of Orange II than that provided by tangential filtration process membranes or unconventional methods, such as microfiltration and ultra-filtration. The literature reports efficiency above 80% for removing azo dyes using these methods [30,31,32]. The results from this study confirmed that the semi-permeable membrane retains solutes [33,34,35].

**Table 3:** Complete assays for removing Orange II related to the proportion of azo dye and albumin bath, contact time, pH before and after filtration.

Assay	Relation dye/albumin v/v (mL)	Contact time (min)	pH before filtration	pH after filtration	Retention time %
1	50/50	60	3.5	3.2	27.84
2	50/100	60	3.5	3.2	33.83
3	50/150	60	3.5	3.2	85.00
4	50/200	60	3.5	3.2	69.57
5	50/250	60	3.5	3.2	77.28
6	25/75	20	3.5	3.9	46.81
7	25/100	20	3.5	3.6	66.67
8	25/125	20	3.5	3.7	13.34
9	25/75	20	3.0	3.0	46.16
10	25/75	20	3.1	3.1	21.96
11	25/75	20	3.2	3.2	21.43
12	25/75	20	3.3	3.3	24.45
13	25/75	80	3.6	3.6	54.77
14	8.5/34	80	3.5	3.5	90.33
15	12/48	60	3.5	3.5	97.15
16	25/75	60	3.5	3.5	99.50

Azo dye/albumin concentration 0.10 g/L.

The Langmuir isotherm has an equilibrium concentration area between the concentrations of the dye (0.1 g/L), depending on the adsorbed mass, and the mass of adsorbent in relation to the concentration in the adsorbed system. These results described the concentration of the azo dye remaining in the water at the adsorption equilibrium point depends on a variety of factors, such as efficiency of interaction of the sorbent and the pH. The literature data are essentially corroborated with the results obtained with the decolorization process [20,21,36].

**Figure 6:**
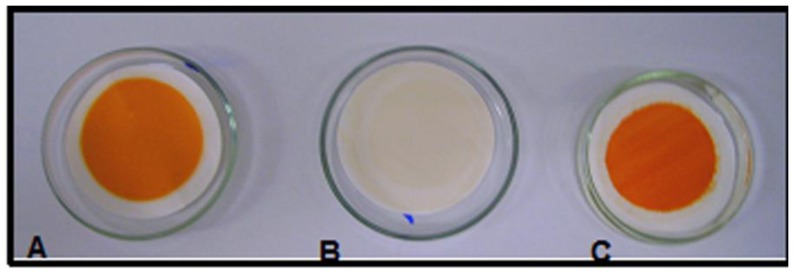
Residual complex formed of Orange II adsorped to albumin using vacuum filtration [Assay 16]. A = residue on cellulose acetate membrane obtained from maximum removal of azo dye/albumin 0.10 g/L (1:3 v/v) at 90 min of incubation, pH 3.0; B = Filtrate of azo dye (0.10 g/L)/water on cellulose acetate membrane; C = Filtrate obtained from azo dye 0.10 g/L cellulose acetate membrane.

A chronic toxicity bioassay was conducted with the microcrustacean *Artemia salina* as the test organism for the toxic azo dye Orange II in order to determine if the species is an appropriate indicator of pollution in aquatic environments.The filtrates that achieved 99.50% and 90.33% removal of azo dye by albumin, in both pH conditions was similar at the start and end of the process. On the other hand, the results showed a higher removal (99.50%) of the proportion of azo dye and albumin 1:3 (v/v). All of these results were attributed mainly to the ionic attraction of albumin associated with Orange II pH [22,23,37,38,39,40] (Figure 6). The microcrustacean *Artemia salina* remained viable when it was exposed to all filtrates after colour removal, and suggests an absence of toxicity. The results indicated that a lethal concentration (LC_50_) is equal to 79 µg/mL of the azo dye Orange II.

## 3. Experimental

### 3.1. Dye, Effluent, Albumin and Membrane

**Figure 7:**
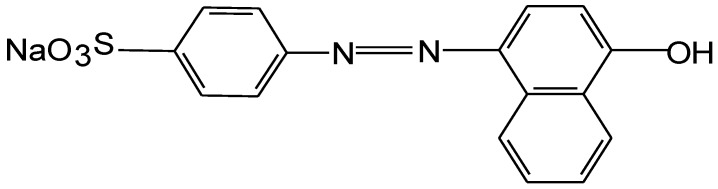
Chemical structure of the azo dye Orange II.

Class II Orange monoazo dye was obtained from Sigma-Aldrich. The molecular mass of Orange II is 350 Da (g/mol), and the polyamide wavelength maximum absorption (−l_max_) is 485 nm. A schematic of the chemical structure of the anionic monoazo species is shown in Figure 7.

The effluent used in this study was simulated in the laboratory using protocols that are typically used in the textile industry when dyeing wool. The purified Orange II was prepared using 0.1 g/L concentration of adsorbate based on the efficiency of dye fixation in the fibre being 80% (source: industrial information).

The protein solution used in the experiment was bovine serum albumin (BSA, Sigma-Aldrich), which was used in a concentration of 0.1 g/L and 0.25 g/L diluted in ultrapure water, pH 11 and was maintained at 5 °C. The membrane used for vacuum filtration was cellulose acetate, which was supplied by Millipore (47 mm diameter; 9.6 cm^2^ area of filtration; 0.2 µm porosity) [41].

### 3.2. Vacuum Filtration Procedure

The tests were performed in batches with distilled water, using the effluent prepared in laboratory with Orange II. The filtration unit consisted of the Millipore kit for vacuum filtration, the membrane filter and a CIENTEC vacuum pump with negative pressure flow of 28 inches of Hg. The interaction between the dye and the albumin was promoted by stirring using a magnetic stir plate (FANEM model 258) at 220 rpm. The experimental processes of separation using the vacuum filtration unit were performed at room temperature (30 °C). The tests were performed using various concentration of albumin to determine the best conditions for adsorption regarding the pH, incubation time for the formation of electrostatic complex and the formation of the permeate.

### 3.3. Colour Reduction Measurement

Colours measurements were performed with a Spectronic Genesis, model 2 UV-visible spectrophotometer. The wavelengths for the measurements were set at the values of the azo dye concentrations and were calculated using an absorbance at 485 nm as per the calibration curve. The percentage of the solute retention time of the membrane was calculated from the readings taken for the samples before and after filtration.

### 3.4. Albumin Determination

The albumin concentration was determined using biuret reagent (Labstest) and was measured on a spectrophotometer (λ = 545 nm).

### 3.5. Experimental Factorial Design

To determine the effects of the azo dye and albumin decolorization, an analysis of variance (ANOVA), with 95% confidence limits was conducted; the results are shown in Table 4. The effects of the variables are graphically illustrated using Pareto charts. A Pareto chart consists of bars with a length proportional to the absolute value of the estimated effects, divided by the standard error. When using the Pareto chart to analyse the effect of variance, estimates are sorted from the highest to the lowest absolute value. The chart includes a vertical line at critical t value for an alpha of 0.05. Effects, for which the bars are smaller than the critical t-value, are not considered to be significant nor do they affect the response variables. The effects may be positive or negative. Statistica^®^ software version 7.0 was used to conduct the ANOVA so as to determine the regression coefficients and produce the graphs [42].

### 3.6. Adsorption Isotherms

The experiments were conducted on a vortexer (200 Hz) at 28 °C. The samples were collected after addition of azo dye albumin over a period of 90 min [41,42,43]. The uptake of the Orange II in the solution was determined by absorbance at 485 nm. All experiments were performed in triplicate.

**Table 4:** Levels of 2^2^ full factorial designs.

	**First full factorial design**		
**Factor**	**Level**		
	**−1**	**0**	**+1**
Azo dye/Albumin0.10 g/L (v/v)	1:3	1:2	1:1
Time of incubation (min)	30	60	90
	**Second full factorial design**		
**Factor**	**Level**		
	**−1**	**0**	**+1**
Azo dye/Albumin0.25 g/L (v/v)	1:3	1:2	1:1
Time of incubation (min)	30	60	90

## 4. Conclusions

The new methodology that was investigated in this study proved to be an innovative and promising adsorption process for removing recalcitrant azo dyes from textile industry wastewater. The high removal rates achieved suggest that the treated effluent may be reused. Furthermore, the new process was reproducible, effective, and low cost and should be considered as a method for treating dyes.
